# Phosphodiesterase-5 Inhibition Alleviates Pulmonary Hypertension and Basal Lamina Thickening in Rats Challenged by Chronic Hypoxia

**DOI:** 10.3389/fphys.2018.00289

**Published:** 2018-03-27

**Authors:** Coline Nydegger, Carla Martinelli, Fabiano Di Marco, Gaetano Bulfamante, Ludwig von Segesser, Piergiorgio Tozzi, Michele Samaja, Giuseppina Milano

**Affiliations:** ^1^Laboratory of Cardiovascular Research, Department of Surgery and Anesthesiology, University Hospital of Lausanne, Lausanne, Switzerland; ^2^Department of Health Science, University of Milan, Milan, Italy; ^3^ASST Santi Paolo e Carlo, Milan, Italy

**Keywords:** pulmonary hypertension, nitric oxide, phosphodiesterase 5, sildenafil, right heart failure, nitrites and nitrates, endothelial NO synthase, pulmonary vascular remodeling

## Abstract

**Background:** Hypoxia represents both an outcome of cardiopulmonary diseases and a trigger for severe pulmonary complications as pulmonary hypertension. Because nitric oxide (NO) is a critical mediator in the development of pulmonary hypertension, the modulators of its downstream function may become target of pharmacological interventions aimed at alleviating the impact of this condition. Here, we investigate the effects of an early administration of phosphodiesterase-5 inhibitor in rats where pulmonary artery hypertension was induced by chronic exposure to hypoxia.

**Methods:** Rats were divided into three groups: normoxic control, hypoxic with no treatments (2 weeks breathing an atmosphere containing 10% oxygen), and hypoxic treated with sildenafil (1.4 mg/Kg per day in 0.3 mL i.p.). After sacrifice, hearts and lungs were removed and harvested for analyses.

**Results:** Sildenafil reduced hypoxia-induced right ventricle hypertrophy without effects in lung hypertrophy, and blunted the increase in right ventricle pressure without effects on left ventricle pressure. Furthermore, the NO-producing systems (i.e., the phosphorylation of the endothelial isoforms of NO synthase that was measured in both myocardial and lung tissues), and the blood NO stores (i.e., the plasma level of nitrates and nitrites) were up-regulated by sildenafil. We did not find significant effects of sildenafil on weight and hemoglobin concentration. Morphological analysis in lung biopsies revealed that 2-week hypoxia increased the frequency of small pulmonary vessels leaving large vessels unaffected. Finally, ultrastructural analysis showed that sildenafil down-regulated the hypoxia-induced increase in the thickness of the pulmonary basal lamina.

**Conclusions:** In this model of pulmonary hypertension, sildenafil contrasts the negative effects of hypoxia on pulmonary vascular and right ventricle remodeling. This action does not only encompass the canonical vasomodulatory effect, but involves several biochemical pathways. Although the human pathological model is certainly more complex than that described here (for example, the inflammatory issue), the potential role of phosphodiesterase-5 for long-term treatment, and perhaps prevention, of pulmonary hypertension is worthy of investigation.

## Introduction

Pulmonary hypertension (PH), a devastating complication of several cardiopulmonary diseases such as chronic heart failure (CHF) and chronic obstructive pulmonary disease (COPD), arises from the progressive narrowing or destruction of the arteries that carry blood from the heart to the lungs. The raise in pulmonary arteries pressure strains the right ventricle (RV) causing hypertrophy and eventually leading to right heart failure. This pathobiology is complicated by hypoxia, which is at the same time an outcome of pulmonary diseases and an established trigger for PH as it was reported, for example, in rats breathing 10% O_2_ for 2 weeks, which develop marked signs of RV hypertrophy (Milano et al., 2011). Impaired nitric oxide (NO) bioavailability is a key feature in most forms of clinical and experimental PH (Giaid and Saleh, 1995; Bueno et al., 2013). Classically, NO is believed to modulate the vascular function through stimulation of smooth muscle cell soluble guanylate cyclase that catalyzes the conversion of guanosine triphosphate into cyclic guanosine monophosphate (cGMP), which lowers cytoplasmic Ca^2+^ and mediates smooth muscle cell relaxation. Pharmacologically, this effect can be made more persistent and intense by selective inhibitors of phosphodiesterase type 5 (PDE5), an enzyme that degrades cGMP into inactive 5′GMP. The best known example of these inhibitors, sildenafil surged as a drug able to alleviate symptoms in several cardiopulmonary diseases including pulmonary arterial hypertension (PAH) (Guazzi and Samaja, 2007) and to correct hypoxia-induced RV hypertrophy (Milano et al., 2011). The underlying scenario reflects a situation where the sildenafil-induced increase in cGMP levels upregulates the phosphorylation of the endothelial isoform of NOS (eNOS), which increases the phosphorylation of Akt, thereby mitigating apoptosis in cardiomyocytes. Although we are far from definite data due to the controversy of available results, in some cases the administration of sildenafil revealed to be useful for the treatment of cardiac heart failure devoid of remarkable adverse effects (Guazzi et al., 2007). The same NO/cGMP pathway is used by the drug riociguat that stimulates the activity of soluble guanylate cyclase independently of NO and acts in synergy with NO to produce anti-proliferative and vasodilatory effects (Lang et al., 2012). However, sildenafil may also have effects not mediated by modulation of vasoconstriction, and in lungs it has been shown to improve pulmonary hemodynamic by modulating the recruitment of bone marrow-derived c-kit+ cells (Favre et al., 2017).

The basal lamina, a layer of extracellular matrix secreted by epithelial cells, acts both as an attachment point for cells and as a permeability barrier. Degenerative pathologies like diabetes mellitus, but also primary respiratory diseases such as asthma and COPD, are known to induce a thickening of the pulmonary basal lamina (Weynand et al., 1999; Liesker et al., 2009), whose thickness represents a compromise between two opposite needs, providing mechanical resistance against excessive pressure buildup, and facilitating the diffusion of gasses across the alveolar-capillary barrier (West and Mathieu-Costello, 1999). This compromise may be overruled in several instances, and hypoxia-induced pulmonary edema has been attributed to stress failure of pulmonary capillaries, which may be due by un-matching the delicate balance between basal lamina strengthening and increased pulmonary hypertension (West et al., 1995). However, direct measurement of the basal lamina thickness as a function of hypoxia is still lacking, consequently it may become difficult to assess whether acute alterations of the thickness and strength of the basal lamina may make part of hypoxia adaptation and pulmonary hypertension development. Aim of this study was to test the impact of sildenafil, a PDE5 inhibitor, on right heart and pulmonary vessels in an experimental model of hypoxia-induced PH.

## Methods

### Animal experiments

We used 42 male 8-week old Sprague-Dawley rats (200–250 g initial nominal weight). Rats were randomly divided into three groups: control, exposed to hypoxia (10% O_2_) for 2 weeks, and exposed to hypoxia and treated with sildenafil (1.4 mg/Kg per day in 0.3 mL i.p.). Each group was composed of two subgroups of 6 rats/each. Rats from the first and second subgroup were used for morphometry/hemodynamics, and biochemical measurements, respectively. Hypoxia was administered in specially designed chambers that enable all treatments, including drug administration and animals handling, avoiding any exposure of animals to atmospheric air (Milano et al., 2002, 2004). Sacrifice was performed 24 h after the last administration. Rats were anesthetized (80 mg/kg xylazine, 100 mg/kg ketasol and 1,500 IU heparin i.p.) in the compensation chamber kept at 10% O_2_. This study was carried out in accordance with the recommendations of the National Institutes of Health (NIH Publication No. 85–23, Revised 1996). The protocol was approved y the Government Veterinary Office (Lausanne, Switzerland; authorization Nr VD2467.1).

### Hemodynamic monitoring

At the end of study, the left ventricle (LV) and right ventricle (RV) systolic pressures (RVSP and LVSP, respectively) were measured by placing a Millar Mikro-Tip conductance catheter (SPR-838, 2F catheter, Millar Instruments Inc., Oxford, UK, coupled to a MPVS Ultra system Millar Instruments, Oxford, UK) as described (Favre et al., 2017). Briefly, anesthetized rats were placed on a heating pad at 37°C and ventilated at 50 cycles·min^−1^ with a tidal volume of 2.5 ml (Harvard Apparatus model 683, Holliston, MA, USA) with either room air or hypoxic atmosphere for normoxic and hypoxic groups, respectively. To evaluate the LVPS, the Millar catheter was introduced into the LV via right carotid artery. To evaluate the RVSP, the chest cavity was opened and the Millar catheter was introduced in the RV cavity using a 24-gauge needle.

### Blood measurements

A blood sample was taken into heparinized tubes after euthanasia from the descending abdominal aorta for measurement of blood hemoglobin (Servomex Oxygen Analyzer 570 A, Zurich, Switzerland) and plasma nitrates and nitrites (colorimetric Griess reaction).

### Animal sacrifice and pulmonary and cardiac hypertrophy

After the hemodynamic measurements, lungs and hearts were perfused en-block with PBS via the RV with efflux through a small opening in the left atrium as reported (Favre et al., 2017). Then, lungs and hearts were placed in PBS solution and kept on ice. After removing the atria from the ventricles, the RV was separated from the LV and the septum (S), blotted dry and weighed to obtain the ratio of RV/(LV+S).

### Pulmonary vascular remodeling

The degree of muscularization of pulmonary arterioles was assessed from immunohistochemical staining of the small pulmonary artery, as previously described (Favre et al., 2017). Briefly, paraffin-embedded lungs were serially sectioned at 8 μm thickness. Following citrate-based antigen retrieval, the sections were blocked with 5% (v/v) goat serum for 1 h. Then, sections were incubated with an antibody against smooth muscle α-actin (α-SMA 1:250, clone 1A4, Sigma-Aldrich) overnight at 4°C, followed by a goat anti-mouse IgG secondary antibody (1/500, DAKO). Pulmonary arterioles were divided by outer vessel diameter in four categories: small (0–50 μm), medium (50–100 μm), large (100–200 μm) and very large (>200 μm) diameters. Pulmonary vascular remodeling was assessed by the percent medial wall thickness. Ten vessels were analyzed for each rat, in seven rats per group per time-point, except for very large (>200 μm) vessels, where the number of analyzed vessels was 2–3. Morphological analyses were conducted in a double-blind method.

### Protein extraction and western blot analysis

In a subset of experiment animals, standard Western blotting analysis was performed using lung and cardiac lysates, as described previously (Favre et al., 2017). The membranes were incubated overnight at 4°C with antibodies against p-eNOS (Ser^1177^, 1:1,000, Cell Signaling Technology) and e-NOS (1:1,000, Santa Cruz Biotechnology, Santa Cruz). Secondary HRP-conjugated antibodies were applied for 1 h at room temperature (1:4,000). Signals were detected by using the enhanced chemiluminescence system (Amersham, Arlington Heights, IL, USA) of a commercial ECL kit. Quantification of the band intensities was performed using NIH ImageJ.

### ELISA

VEGF and VEGF-R1 protein levels were quantified in lung lysates by ELISA (ELISA kit, Assay Designs, Inc., MI). All assays were made in duplicate and performed according to the manufacturer's instruction.

### RT-PCR

Total RNA of lung tissues was extracted using the TRIzol method as described (Favre et al., 2017). The relative amount of mRNA expression for vascular endothelial growth factor (VEGF) was represented using the 2-ΔΔCt value. The primer pairs for VEGF (109 bp) were designed (sense: 5′-AGTACCTGTTCTGGCTAATGG-3′; anti-sense: 5′-TCACTTTCGTGCGCTCGTAG-3′) and for the housekeeping gene GAPDH (359 bp) were (forward) 5′-TGAAGGTCGGTGTGAACGGATTTG-3′ and (reverse) 5′-GGCGGAGATGATGACCCTTTTG-3′, respectively.

### Transmission electron microscopy (TEM)

Lung biopsies were fixed in 2.5% (v/v) glutaraldehyde dissolved in 0.13 M phosphate buffer (pH 7.2–7.4). Five specimens for each biopsy were post-fixed in 1% OsO_4_, dehydrated in ethanol plus propylene oxide and embedded in epoxy resin. Ultrathin 50–60 nm sections were routinely counterstained with uranyl acetate (10 min, Merck, Darmstadt, Germany) and lead citrate (5 min, Merck, Darmstadt, Germany), and examined with a JEM 1010 (Jeol, Tokyo, Japan) electron microscope. To determine the median basal lamina thickness, the Marquez simplified method was applied in printed 18 × 24 cm micrographs taken at 24,000 × magnification using 10 areas at a fixed 4 mm distance from one to another. The average thickness measured in the 10 areas was considered as the final value. To determine the mitochondrial size, the areas of 30 randomly selected mitochondria for each condition was calculated.

### Statistics

Data are expressed as mean ± SEM. To measure the effects of hypoxia and sildenafil, we performed one-way analysis of variance followed by the Tukey's multiple comparison test if significant. The significance level was set at *P* = 0.05.

## Results

### Whole animal data

All animals survived the experimental protocol without signs of discomfort. Whole animal data are reported in Table 1. Exposure to hypoxia for 2 weeks decreased body weight. Likewise, hypoxia increased blood hemoglobin, hematocrit and red cell count. No changes were observed in heart weight, but hypoxia increased the wet weight in both lungs. None of these variables was affected by sildenafil.

**Table 1:** Whole animal data expressed as mean ± SEM.

	**Normoxia**	**Hypoxia**	**Hypoxia + sildenafil**	**1-way ANOVA**
Body weight initial, g	248.1 ± 3.0	246.5 ± 8.5	254.5 ± 9.1	NS
Body weight final, g	340.3 ± 7.9	252.2 ± 10.4^§^	271.8 ± 5.1^§^	<0.0001
Heart weight, g	1.303 ± 0.047	1.383 ± 0.058	1.161 ± 0.054	0.0484
Lungs weight, g	1.545 ± 0.071	1.990 ± 0.058^§^	1.957 ± 0.063^§^	<0.0001
Hematocrit, %	50.7 ± 1.5	66.0 ± 2.4^§^	64.7 ± 1.3^§^	<0.0001
Hemoglobin, g/L	158.18 ± 3.58	200.25 ± 7.25^§^	201.53 ± 5.15^§^	<0.0001
RBC/fl	8.65 ± 0.36	10.45 ± 0.43^§^	10.05 ± 0.29^§^	<0.0001

§ *P < 0.05 vs. normoxia (Tukey's multiple comparison test). No significant differences have been observed between hypoxia and hypoxia + sildenafil*.

### Sildenafil reduced hypoxia-induced right ventricle hypertrophy without effects in lung morphology

**Figure 1:**
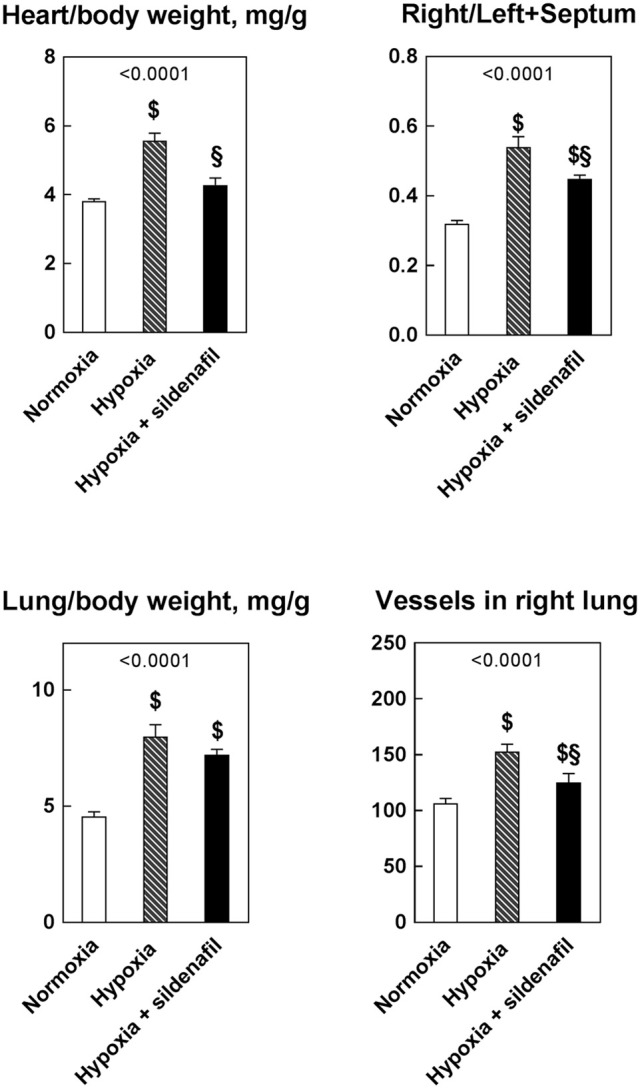
Effects of 2-week hypoxia (10% O_2_) and hypoxia + sildenafil on the heart/body weight ratio, right ventricle hypertrophy, lung/body weight ratio, and number of vessels in the right lung. Data are expressed as mean ± SEM. The 1-way ANOVA value is reported for each variable. ^$^*P* < 0.05 vs. normoxia, ^§^*P* < 0.05 between hypoxia and hypoxia + sildenafil (Tukey's multiple comparison test).

Although the heart weight was apparently unaffected by neither hypoxia, nor sildenafil, the simultaneous decrease in body weight led to varied heart/body weight ratios, which is an index of myocardial hypertrophy (Figure 1). This ratio increased markedly in untreated rats upon hypoxia, but the administration of sildenafil markedly blunted this effect. The RV/(LV+S) ratio is a reliable index of RV hypertrophy. This ratio increased markedly after 2-week hypoxia, but the increase was less in sildenafil-treated rats. The lung/body wet weight was also markedly increased by hypoxia, and sildenafil was unable to alleviate this increase. Likewise, the number of vessels in the right lung increased during hypoxia and sildenafil corrected this increase.

### Sildenafil blunted the increase in right ventricle pressure without effects on left ventricle pressure

**Figure 2:**
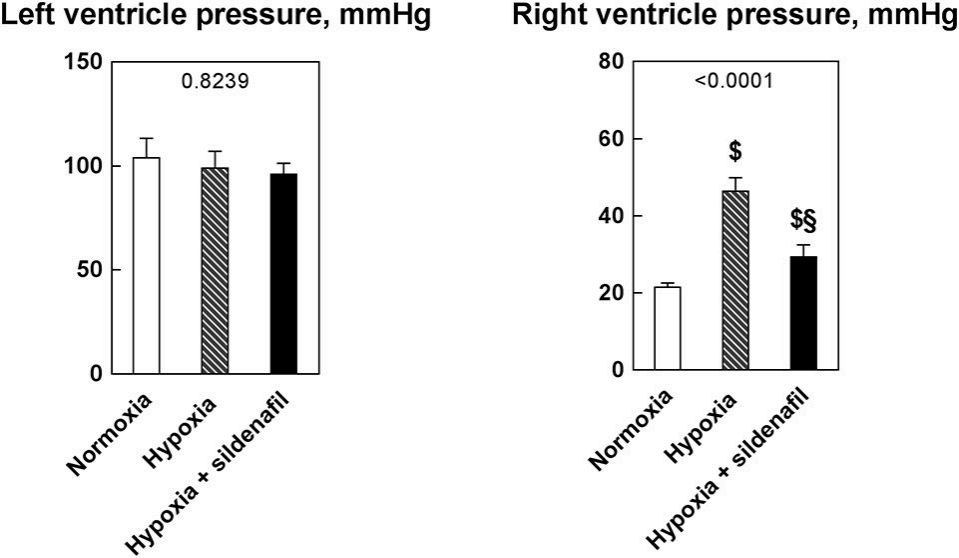
Effects of 2-week hypoxia (10% O_2_) and hypoxia + sildenafil on the pressures developed by the left and right ventricles. Data are expressed as mean ± SEM. The 1-way ANOVA value is reported for each variable. ^$^*P* < 0.05 vs. normoxia, ^§^*P* < 0.05 between hypoxia and hypoxia + sildenafil (Tukey's multiple comparison test).

Figure 2 shows the left and right ventricle pressure as measured as explained in the Materials and Methods section. The pressure developed by the left ventricle was not affected by neither hypoxia nor sildenafil. By contrast, the pressure developed by the right ventricle was increased by hypoxia, indicative of PAH development. This increase was markedly blunted in sildenafil-treated rats.

### Two-week hypoxia increases the frequency of small pulmonary vessels leaving large vessels unaffected

**Figure 3:**
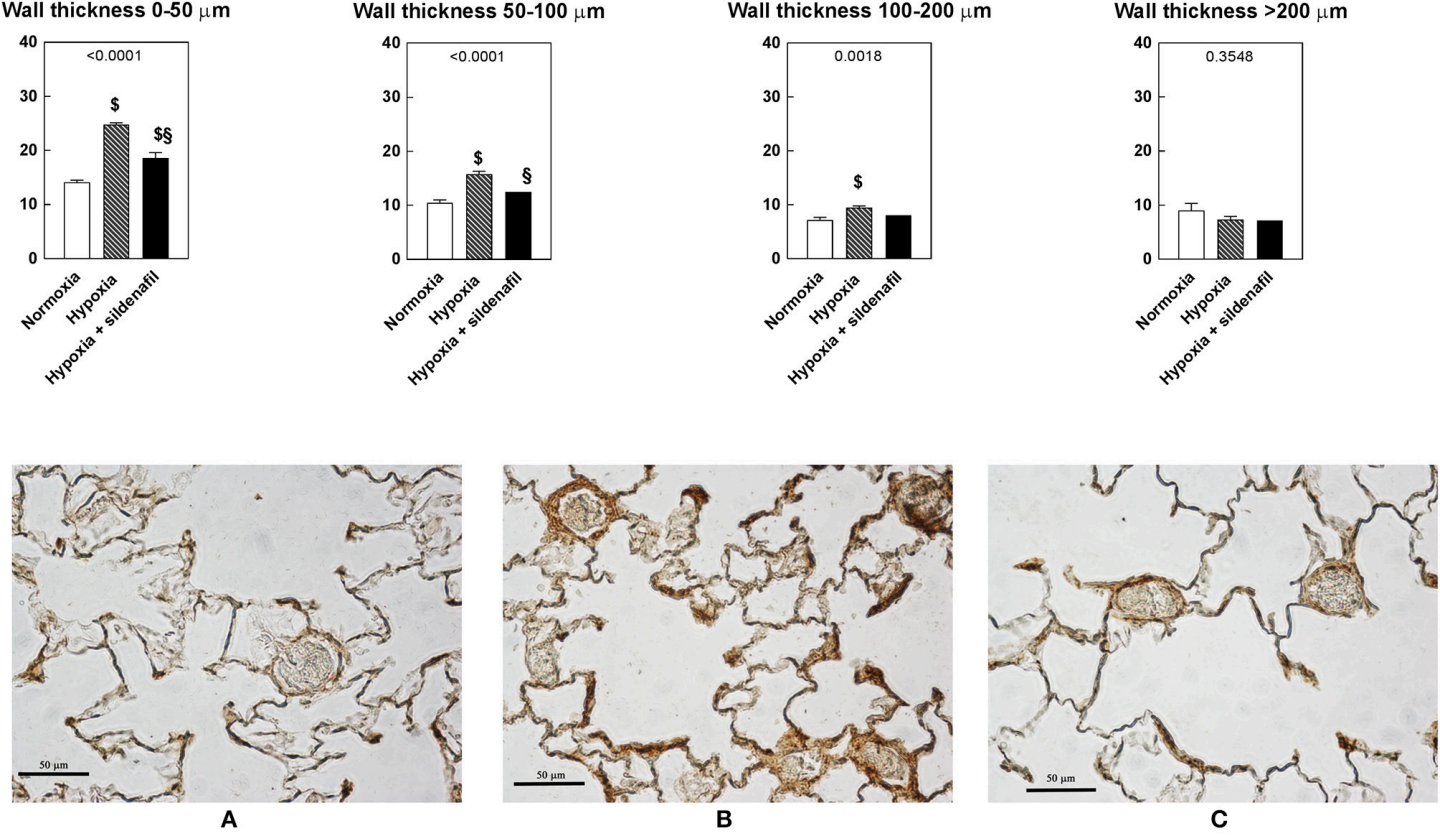
Effects of 2-week hypoxia (10% O_2_) and hypoxia + sildenafil on the wall thickness of small (0–50 μm), medium (50–100 μm), large (100–200 μm), and very large (>200 μm) pulmonary vessels determined as explained in the Materials and Methods section. The lower row shows representative images taken from each group. Notations **(A-C)** refer to normoxia, hypoxia, and hypoxia + sildenafil, respectively. The bar represents 100 μm. Data are expressed as mean ± SEM. The 1-way ANOVA value is reported for each variable. ^$^*P* < 0.05 vs. normoxia, ^§^*P* < 0.05 between hypoxia and hypoxia + sildenafil (Tukey's multiple comparison test).

As shown in Figure 1, hypoxia increased the number of pulmonary vessels. To ascertain whether this increase was shared to both newly formed and mature vessels, we measured the frequency of vessels in four categories of wall thickness, arbitrarily divided into small (0–50 μm), medium (50–100 μm), large (100–200 μm), and very large (>200 μm) diameters (Figure 3). It appears that the effect of hypoxia was more pronounced for small vessels and progressively diminished with the vessels diameter. As a result, the anti-hypoxic effect of sildenafil was more marked in small than in large vessels. The frequency of very large vessels was unaffected by either hypoxia or sildenafil.

### NO-producing systems are up-regulated by sildenafil

**Figure 4:**
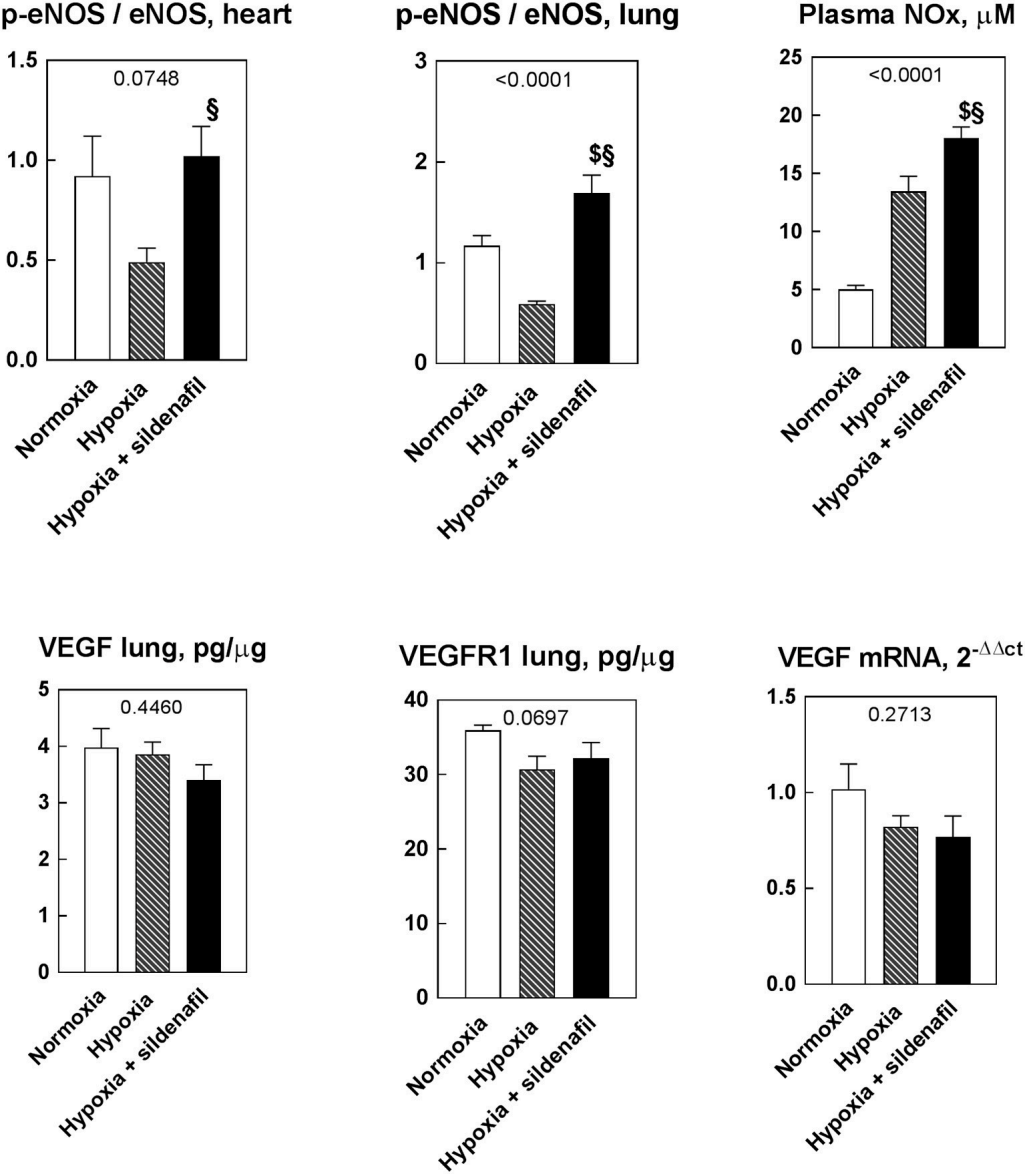
Effects of 2-week hypoxia (10% O_2_) and hypoxia + sildenafil on the phosphorylation and activation of NO-generating enzymes as well as on the level of nitrites and nitrates in plasma **(Upper row)**, and VEGF-related parameters **(Lower row)**. Data are expressed as mean ± SEM. The 1-way ANOVA value is reported for each variable. ^$^*P* < 0.05 vs. normoxia, ^§^*P* < 0.05 between hypoxia and hypoxia + sildenafil (Tukey's multiple comparison test).

The p-eNOS/eNOS ratio highlights the activation of NO-producing enzymes. In both heart and lung biopsies, hypoxia decreased this ratio, while sildenafil promoted eNOS phosphorylation (Figure 4). The plasma level of nitrates and nitrites (NOx) marks the NO stores level. While hypoxia produced an increase in plasma NO stores, sildenafil was able to further increases it. However, neither hypoxia nor sildenafil were able to affect none of the factors linked to the vascular endothelial growth factor (VEGF). Indeed, the protein expression of VEGF and VEGF-R, as well as VEGF mRNA remained constant in the three groups.

### Sildenafil efficiently down-regulates the hypoxia-induced increase in the thickness of the pulmonary basal lamina

**Figure 5:**
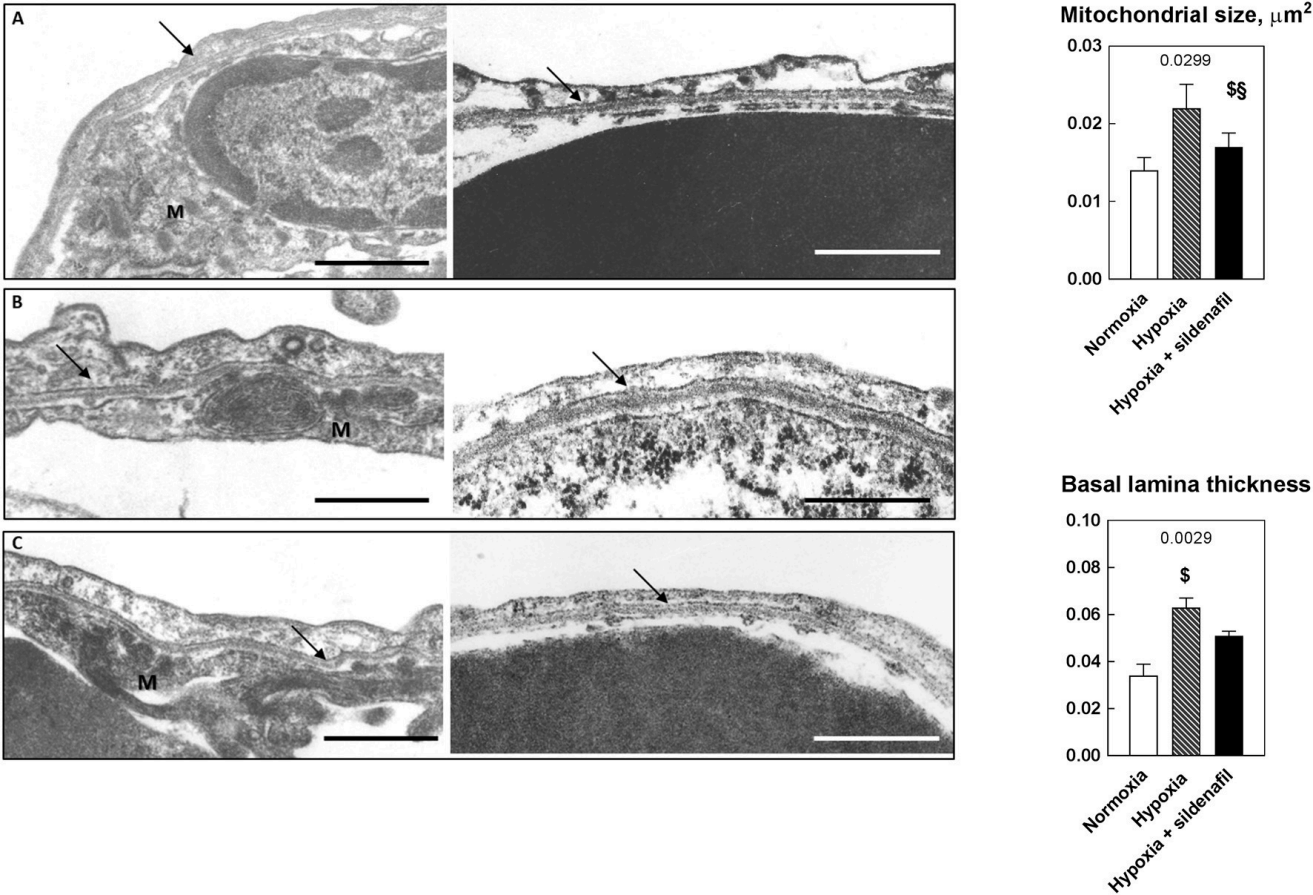
Effects of 2-week hypoxia (10% O_2_) and hypoxia + sildenafil on mitochondrial size and the thickness of the pulmonary basal lamina. Two representative micrographs for each group are displayed. Notations **(A-C)** refer to normoxia, hypoxia, and hypoxia + sildenafil, respectively. Arrows indicate the basal lamina, while the notation M indicates the mitochondria (scale bars = 0.5 μm). Averaged data on the right panel are expressed as mean ± SEM. The 1-way ANOVA value is reported for each variable. ^$^*P* < 0.05 vs. normoxia, ^§^*P* < 0.05 between hypoxia and hypoxia + sildenafil (Tukey's multiple comparison test).

Figure 5 shows some representative electron microscope images highlighting the effects of 2-week hypoxia (10% O_2_) and sildenafil on mitochondrial size and the thickness of the basal lamina. Averaged data obtained in all available samples are shown on the right panel. As expected, hypoxia increased the mitochondrial size, and sildenafil contrasted this effect. Likewise, while hypoxia increased the thickness of the basal lamina, sildenafil down-regulated this increase.

## Discussion

In this study, we report some cardiopulmonary responses to 2-week chronic hypoxia. The severity of the hypoxia challenge, 10% O_2_, is known to induce molecular and cellular alterations without becoming lethal even after 4 weeks in mice (Terraneo et al., 2017). In the rat, breathing 10% O_2_ for 2 weeks impairs the myocardial tolerance to ischemia/reperfusion (Milano et al., 2002), induces RV hypertrophy (Corno et al., 2004), impairs ${\text{K}}_{\text{ATP}}^{+}$ channels opening (Milano et al., 2004), activates hypoxic signaling and apoptosis (Bianciardi et al., 2006), modulates differentially the expression of mitogen-activated protein kinases (Caretti et al., 2007) and increases the immunoreactivity against the 70 kDa heat shock proteins (Tarricone et al., 2008). Furthermore, 2-week hypoxia also reduces the myocardial p-eNOS/eNOS ratio while increasing plasma NOx in a mechanism resembling that underlying intermittent hypoxia (Milano et al., 2010, 2011). Here, we show that most of the hypoxia-induced changes that regard NO handling in the lungs may be strongly modulated by sildenafil.

Despite its short plasma half-life of sildenafil of 4–6 h (Boolell et al., 1996), the selected dose and timing for sildenafil administration was already shown to be sufficient to enable sildenafil to hit its target by inducing a x4 increase in cGMP in heart tissue (Milano et al., 2011). Sildenafil was shown to protect tissue by elevating the protein level of both iNOS and eNOS (Salloum et al., 2003). The activation of the NO-producing system by eNOS Ser^1177^ phosphorylation was reported to be a key step through which sildenafil exerts cardioprotective effects in chronically hypoxic hearts (Baker et al., 2001) by reducing apoptosis, enhancing protein kinase B activation (Milano et al., 2011), down-regulating intracellular calcium by mitochondrial K^+^ATP channels opening (Fitzpatrick et al., 2005), and attenuating the recruitment of bone-marrow-derived c-kit(+) cells (Favre et al., 2017). Remarkably, we were unable to document a similar effect on VEGF and VEGF-R1 expression that were reviewed to be upregulated by sildenafil in an attempt to improve angiogenesis to restore the hypoxia-challenged viability (Liu and Simon, 2004). However, the situation in lungs that are exposed directly to atmospheric air may be quite different from that in other internal organs where angiogenesis is the key way to transfer the oxygen brought by capillary blood. Because sildenafil did not affect the blood hemoglobin concentration, the increased activation of eNOS reflects into higher plasma NO stores or NOx.

The total number of vessels in both lungs appear to follow the same pattern described for eNOS phosphorylation and NO stores: whereas exposure to hypoxia increases the value, the administration of sildenafil can markedly reduce, and sometimes blunt, such increase. However, the changes in size distribution of pulmonary vessels is dependent on the vessel diameter. Small pulmonary vessels are strongly affected by hypoxia and by sildenafil. By contrast, larger pulmonary vessels are less affected by hypoxia and sildenafil. If the size of the pulmonary vessels is associated with their degree of maturation, then this may be attributed to the vessel maturation process. The thickness of the basal lamina may be another key to understand the effects of PDE-5 inhibition in lungs. As mentioned above, the pulmonary blood-gas barrier represents a compromise between two opposite needs: a reduced thickness favors oxygen diffusion, while its increase contributes to strengthen the barrier. If hypoxia involves high RVSP, and sildenafil reduces it, this translates into less demand to improve pulmonary resistance against circulatory stress by tightening the basal lamina, thus favoring oxygen diffusion. As such, sildenafil may be considered an “anti-hypoxic” drug.

PH is an important complication in the natural history of cardiopulmonary diseases, such as COPD. Its presence is associated with reduced survival and greater use of healthcare resources. In COPD, pulmonary vascular remodeling, due to inflammation and/or hypoxia, affects small and precapillary arteries, and has been identified at different degrees of disease severity. Patients with end-stage COPD and PH show deposition of longitudinal muscle, fibrosis and elastosis that enlarge the intima in pulmonary muscular arteries (Barberà and Blanco, 2009). Impairment of endothelial function may be associated with or result from changes in the expression or balanced release of vasoactive mediators with vasodilator properties, such as NO or prostacyclin, and mediators with vasoconstrictive properties, such as endothelin-1 or angiotensin. Indeed, eNOS expression in pulmonary arteries, which is diminished in patients with idiopathic PAH (Giaid and Saleh, 1995) is also reduced in COPD patients (Yang et al., 2012) and in smokers without airflow obstruction (Barberà et al., 2001). In COPD, pulmonary vasodilation can lead to deterioration of gas exchange due to the high ventilation/perfusion ratio mismatch areas. Inhaled prostanoids may acutely reduce pulmonary arterial pressure while largely maintaining gas exchange; however, long-term clinical trials have not been reported. Robust data for the clinical beneficial effect of endothelin receptor antagonists (ERAs) on pulmonary hemodynamics and exercise tolerance in COPD with pulmonary hypertension are lacking. Finally, there is definitely a lack of evidence of a long-term clinical beneficial effect of PDE5 inhibitors in COPD patients (Seeger et al., 2013), even if a recently published study demonstrated that treatment with sildenafil is able to reduce pulmonary vascular resistance and improve the BODE index (a multidimensional grading system that predict mortality in COPD) and quality of life, without a significant effect on gas exchange, in a cohort of patients with severe pulmonary hypertension and COPD (Vitulo et al., 2017). In this context, the result of the present study, which demonstrates that sildenafil affects the negative effects of hypoxia on right ventricle through its impact on NO-pathway, may be of interest because one can speculate a protective effect of sildenafil, or other drugs active on the NO-pathway such as riociguat, on vessels remodeling leading and pulmonary hypertension and right ventricle hypertrophy.

## Conclusions

In the described model of pulmonary hypertension induced by chronic exposure to hypoxia, PDE-5 inhibition by sildenafil contrasts the negative effects of hypoxia on pulmonary and right ventricle remodeling. This action does not only encompass the canonical vasomodulatory effect, but involves several cell pathways. Although the human pathological model is certainly more complex than that described here (for example, by including marked pro-inflammatory issues), PDE-5 inhibition may become an appreciable target for long-term treatment of pulmonary hypertension, and perhaps also for the prevention of this debilitating disease.

## Ethics statement

Experimental protocols conformed to Swiss law. The local ethical committee for animal research (Service de la Consommation et des Affaire Vétérinaires, SCAV) approved the protocol (authorization VD2467.1).

## Author contributions

CN performed the experiments and the statistical analysis. CM performed the TEM analyses and participated to the discussion of the results of this manuscript. FD contributed for the clinical impact of the described data and participated to the discussion of the results of this manuscript. GB contributed for the TEM analyses. LvS and PT participated to the discussion of the results of this manuscript. MS performed the statistical analyses, participated to the discussion of the results of this manuscript and wrote the manuscript. GM supervised all the phases of this study.

### Conflict of interest statement

The authors declare that the research was conducted in the absence of any commercial or financial relationships that could be construed as a potential conflict of interest.

## Abbreviations

PD: chronic obstructive pulmonary disease.
eNOS: endothelial isoform of NO synthase
LV: left ventricle
LVSP: LV systolic pressure
NO: nitric oxide, NOx, nitrates and nitrites.
PAH: pulmonary arterial hypertension
PDE-5: phosphodiesterase-5
p-eNOS: phosphorylated isoform of eNOS
PH: pulmonary hypertension
RV: right ventricle
RVSP: RV systolic pressure
TEM: transmission electron microscope
VEGF: vascular endothelial factor
VEGFR1: receptor of VEGF
